# When Mind Hurts the Skin: A Rare Case of Dermatitis Artefacta in an Epileptic Female

**DOI:** 10.1002/ccr3.73411

**Published:** 2026-08-31

**Authors:** Maaz ullah, Tayyeb Ali, Muhammad Hassaan Javaid, Muddassir Khalid, Naveed Ahmad, Farooq Haider, Okasha Tahir, Munazza Iqbal, Sadia Afridi, Ejaz Ali, Fred Segawa

**Affiliations:** ^1^ Department of Medicine Bannu Medical College Bannu Pakistan; ^2^ Department of Medicine Gomal Medical College Dera Ismail Khan Dera Ismail Khan Pakistan; ^3^ Department of Medicine Shifa College of Medicine Islamabad Islamabad Pakistan; ^4^ Department of Medicine Nishtar Medical University Multan Pakistan; ^5^ Department of Medicine Khyber Medical College Peshawar Peshawar Pakistan; ^6^ Department of Medicine Khyber Girls Medical College Peshawar Pakistan; ^7^ Department of Medicine Chengde Medical University Chengde China; ^8^ Department of Medicine Makerere University College of Health Sciences Kampala Uganda

**Keywords:** dermatitis artefacta, epilepsy, psychodermatological disorder

## Abstract

DA is an uncommon psychocutaneous disorder in which the patient intentionally induces lesions to satisfy a subconscious psychological need and, at the same time, denies self‐harm. DA typically presents as bizarre, well‐demarcated lesions in accessible areas. Diagnosis is based on the suspicion and exclusion of organic causes. A 27‐year‐old married woman presented with painful, slow‐healing ulcers on the dorsum of both hands and forearms for 2 months, along with body pain and progressive hair loss. The patient had epilepsy, which was controlled with sodium valproate and carbamazepine, although she had occasional psychogenic non‐epileptic seizures (PNES). She denied systemic symptoms, like fever or arthralgia. Examination revealed irregular punched‐out ulcers with erythematous borders, crusting, and post‐inflammatory hyperpigmentation and infection or signs of systemic illness. Investigations revealed leukocytosis (TLC 19.1 × 10^9^/L), while the brain CT was normal. Skin biopsy revealed epidermal ulceration, necrosis, and mixed inflammatory infiltrates without vasculitis or pathogenic organisms. The patient was admitted to the hospital to manage her lesions when experiencing anxiety attacks, thereby confirming DA. This case represents the typical features of DA: self‐inflicted geometric lesions on accessible sites in a young woman with psychological stressors. The coexistence of epilepsy and pseudoseizures reflects a psychoneurological interaction whereby chronic illness and emotional instability may be the instigators of maladaptive behavior. Management requires a multidisciplinary approach involving dermatology, psychiatry, and neurology. Supportive topical therapy and cognitive‐behavioral therapy resulted in better emotional regulation, adequate sleep, and ulcer healing. Empathetic, integrated management ensures recovery and prevents the recurrence of DA.

## Key Clinical Message

Dermatitis artefacta should be suspected in patients with bizarre, well‐demarcated lesions on accessible body sites after exclusion of organic dermatoses. Recognition of associated psychiatric disorders, including psychogenic non‐epileptic seizures in patients with epilepsy, is essential, as successful treatment relies on empathetic multidisciplinary care integrating dermatological, neurological, and psychiatric management.

## 1 Introduction

Dermatitis artefacta (DA) is a psychocutaneous disorder where patients cause skin lesions to satisfy some internal psychological needs but deny their psychological involvement [1]. It affects young adults and is strongly female‐dominant. It is rarely diagnosed but has a prevalence of 6.7%. Psychiatric comorbidities, such as anxiety and personality disorders, are often correlated, making diagnosis and management increasingly difficult [2, 3].

Many cases are only discovered after several unnecessary tests or when the patient's behavior creates suspicion [4]. Treatment focuses on the management of psychiatric comorbidities and supportive dermatological care [5]. We therefore present a case of a 27‐year‐old female with a history of deep skin lesions and epilepsy.

This case report conforms to the CARE checklist for medical case reports.

## 2 Case History

**FIGURE 1:**
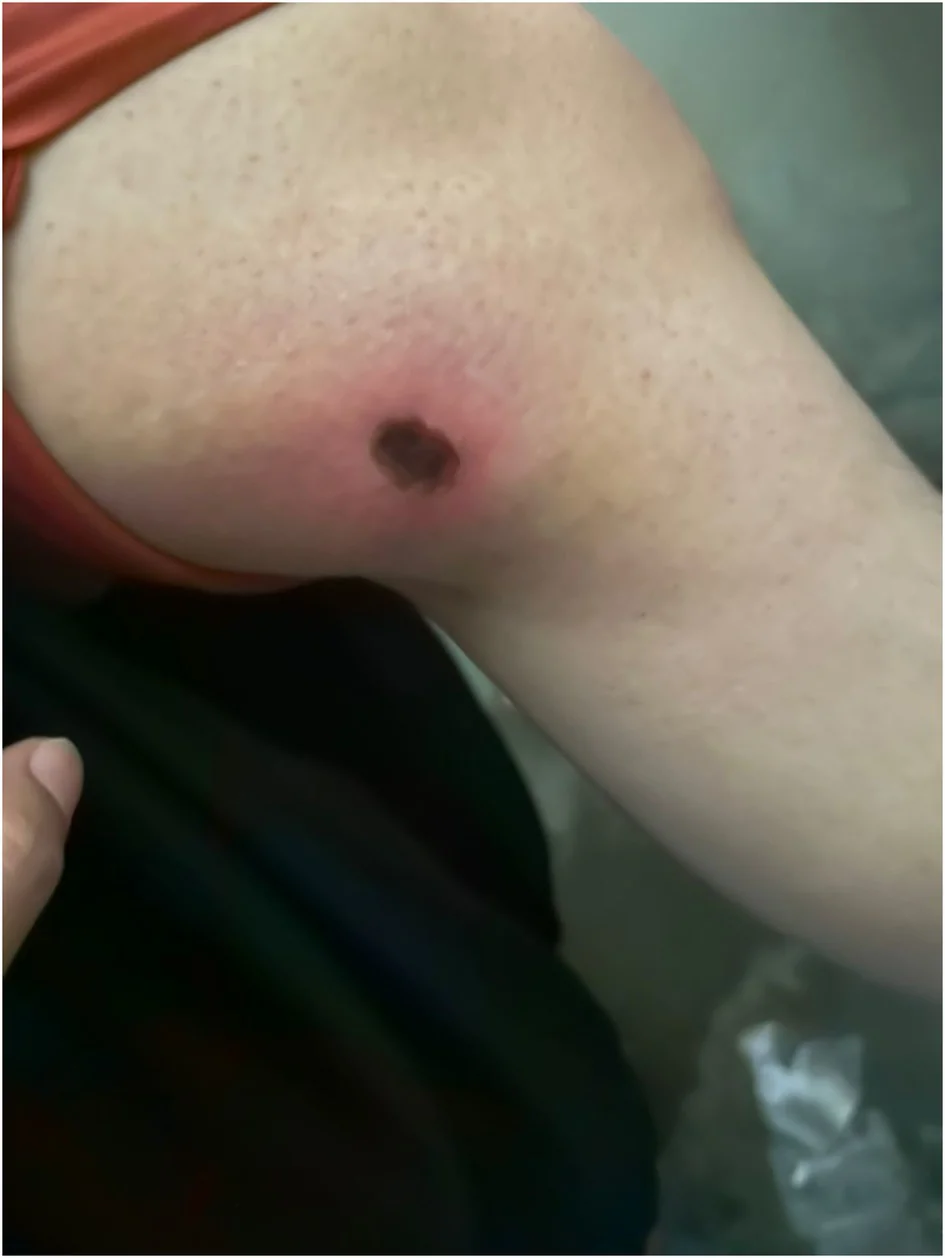
A lesion on the skin caused by the patient.

A 27‐year‐old woman presented with a history of several deep skin lesions on the arms and hands for 2 months, as shown in Figure 1. The lesions were followed by pain, oozing, and a healing delay. She complained of generalized body pain and progressive hair loss for the same period with no history of fever, photosensitivity, or arthralgia. The patient had a known diagnosis of epilepsy and was on chronic medication with Sodium Valproate 500 mg twice a day and Carbamazepine 200 mg twice a day. Along with the confirmed epileptic seizures, she had also had some episodes that clinically met the criteria for psychogenic non‐epileptic seizures (PNES). The distinguishing feature of these seizures was their preservation of consciousness, lack of tongue biting, incontinence of urine, as well as absence of postictal confusion, which made epileptic seizures less probable. The neurological examination, including brain CT, did not show any new lesions, while psychological examination revealed severe anxiety, emotional upset, sleep disturbance, and maladaptive coping mechanisms supporting the diagnosis of PNES occurring alongside established epilepsy.

Her obstetric history was notable for two second‐trimester losses of pregnancy and one full‐term loss. She had one living child. There was no psychiatric illness, autoimmune disease, or chronic dermatologic disease in the family history. The patient reported that her lesions were initially small, whitish vesicobullous eruptions that ruptured spontaneously later to cause oozing and the development of ulcers. The ulcers became larger when she scratched them, and she confessed to frequent manhandling of the lesions because of intense anxiety and restlessness. Personal history was a disturbed sleep pattern and episodes of low mood and emotional instability, especially after stressful situations.

Differential Diagnosis:

1. Pyoderma Gangrenosum: A neutrophilic dermatosis characterized by rapidly enlarging, painful ulcers with undermined violaceous borders, often associated with systemic diseases like IBD or rheumatoid arthritis.
2. Cutaneous Vasculitis: Presents with palpable purpura or necrotic ulcers; however, the biopsy in this case specifically ruled out vascular inflammation.
3. Factitial Panniculitis: An artificial inflammation of the subcutaneous fat caused by the injection of foreign substances or mechanical trauma, presenting as localized nodules or ulcers.
4. Ecthyma: A deep form of impetigo caused by *Streptococcus* or *Staphylococcus* that produces “punched‐out” ulcers; typically responds quickly to antibiotics, unlike the slow‐healing lesions in DA.
5. Delusional Parasitosis: A related psychocutaneous disorder where patients induce skin damage (the “matchbox sign”) due to a firm, false belief that they are infested with organisms.

### 2.1 Investigations

Examination revealed several well‐defined, punched‐out ulcers found mainly on the dorsum of both hands and forearms. The ulcers were irregularly sized and shaped, with erythematous borders, serous crusting, and hemorrhagic eschar sites. The overlying skin was characterized by erythematous papules, healing scars, and post‐inflammatory hyperpigmentation. There were no features of infection, lymphadenopathy, or systemic illness. General physical examination was not remarkable, other than mild fatigue and an anxious demeanor during the interview. The brain CT scan was normal. Complete Blood Count (CBC) was performed, which showed raised TLC levels, which were 19.1 × 10^9^/L.

A skin biopsy showed epidermal ulceration with necrosis, fibrin deposition, and dense mixed inflammatory infiltrate composed predominantly of lymphocytes and neutrophils in the dermis. The biopsy showed the absence of pathogenic organisms and no evidence of vasculitis or specific dermatoses. These histopathological findings, along with the clinical and behavioral presentation of patients, were consistent with Dermatitis Artefacta.

### 2.2 Management

The patient was treated using a multidisciplinary strategy with dermatology, psychiatry, and neurology teams. She was given supportive dermatologic treatment, such as topical antibiotics and emollients, to help heal the ulcers and avoid secondary infection.

Her antiepileptic medications (Sodium Valproate and carbamazepine) were maintained, and seizure activity was closely monitored. Psychiatric evaluation identified features consistent with underlying psychological distress as a cause for self‐inflicted skin injury. She was initiated on psychotherapy, with a focus on cognitive‐behavioral therapy (CBT) to manage stress and maladaptive behaviors. The patient was counseled about the psychogenic origin of her lesions.

### 2.3 Outcomes and Follow‐Up

The patient was re‐evaluated at 8 weeks into the treatment plan involving various health professionals. Adherence to topical care, antiepileptic drug therapy, and cognitive‐behavioral sessions remained intact throughout this period. In terms of clinical findings, no further cases of self‐inflicted skin injury had been found; additionally, previous ulcers continued to heal with a significant improvement in erythema and crusting; the greatest ulcer had healed from 3.0 × 2.5 to 0.8 × 0.6 cm. Improvement in the quality of sleep and a lessening of anxiousness has been reported by the patient and her relatives. There have been no occurrences of the disease recurrence throughout the observation period. Treatment was well tolerated, with no adverse effects related to psychotherapy or topical medications.

## 3 Discussion

The demographic profile of our patient aligns closely with the typical DA presentation, which exhibits a strong female predominance and often begins in young adulthood [6, 7]. The location of the lesions on the easily accessible dorsum of the hands and forearms is a classic feature, as patients typically injure areas within their comfortable reach [8].

The morphology of the lesions, well‐defined, punched‐out ulcers, is highly suggestive of artefactual disease. These bizarre, non‐anatomical patterns are often the first visual clue leading a clinician to suspect DA [8, 9]. Emotional stress, social isolation, and chronic illness may serve as triggers for self‐inflicted behaviors [9]. The patient's history of reproductive losses and disturbed sleep pattern might have contributed to her underlying emotional dysregulation and maladaptive coping mechanisms.

Psychogenic non‐epileptic seizures (PNES) refer to episodic events that mimic epileptic seizures but do not have any abnormal epileptiform electroencephalographic activity and are thought to be a form of functional neurological symptom disorder. It is possible for patients suffering from epilepsy to have PNES concurrently, making diagnosis and treatment difficult. In this case, the absence of characteristic epileptic features from several seizure events along with psychological issues that were shown through a psychiatric workup to have anxiety disorders helped establish PNES rather than uncontrolled epilepsy. The overlap between the two made her emotionally unstable and led to her dermatitis artefacta [10, 11].

The convergence of clinical, histological, and behavioral evidence supported the diagnosis in this case as shown in Table 1. Histopathology in DA is typically nonspecific, as noted in our patient, showing ulceration, necrosis without any diagnostic markers of vasculitis or autoimmune disease. Lesion age and repeated manipulation can influence microscopic findings [12]. The critical diagnostic confirmation came from the patient's own admission of frequent manhandling of the lesions because of intense anxiety and restlessness [13, 14].

**TABLE 1:** Differential diagnosis table of dermatitis artefacta.

Diagnosis	Clinical features	Histopathology	Reason excluded
Dermatitis artefacta	Geometric, bizarre ulcers on accessible sites; inconsistent history	Non‐specific ulceration and inflammation	Clinical behavior and admission of self‐inflicted injury supported diagnosis
Pyoderma gangrenosum	Rapidly progressive painful ulcers with undermined violaceous borders	Dense neutrophilic infiltrate	No characteristic borders or associated systemic disease
Cutaneous vasculitis	Palpable purpura, necrotic ulcers	Leukocytoclastic vasculitis with vessel wall destruction	No vasculitis on biopsy
Factitial panniculitis	Deep nodules or ulcers after trauma/injection	Fat necrosis and panniculitis	Histology inconsistent
Ecthyma	Painful crusted ulcers caused by bacterial infection	Epidermal ulceration with abundant bacteria and acute inflammation	No organisms on biopsy; poor response pattern inconsistent with bacterial infection
Delusional parasitosis	Excoriations due to fixed infestation belief	Usually nonspecific	No delusional belief of infestation

Ecthyma was another significant differential in our patient based on the punched‐out appearance of the ulcers. Several factors, however, made the diagnosis of ecthyma unlikely. Ecthyma usually manifests itself as infectious ulcers caused by *Streptococcus pyogenes* or *Staphylococcus aureus* infections that usually involve surrounding cellulitis, microbiologically positive findings, and prompt improvement with antibiotic treatment. Our patient had lesions localized to easily accessible parts of the body, did not have constitutional symptoms, had no evidence of infection around the skin lesions, and no pathogens were found upon skin biopsy. More importantly, the fact that the lesions persisted during stressful periods and stopped after psychiatric treatment suggested dermatitis artefacta [1, 15, 16].

This case is particularly notable for its complex comorbidity with epilepsy. This coexistence of a documented neurological disorder and a conversion or factitious disorder highlights the intricate interplay between organic brain disease and psychopathology. The psychological burden of a chronic neurological condition, compounded by a tragic obstetric history of multiple pregnancy losses, likely created a fertile ground for profound psychological distress [6, 17].

Management requires a multidisciplinary and empathetic approach [13, 17]. Cognitive‐behavioral therapy (CBT) targeted the core issues of stress, emotional dysregulation, and maladaptive behavior. Pharmacological treatment includes supportive use of selective serotonin reuptake inhibitors (SSRIs) in resistant cases, although our patient showed improvement primarily with psychotherapy and counseling. Gerlero et al. emphasized the importance of continuity of care and family involvement to reduce relapse risk [5]. The improved sleep, reduced lesion formation, and progressive healing at follow‐up validate this approach [18].

## 4 Conclusion

This case serves as a poignant reminder that the skin can be a canvas for internal psychological turmoil. Dermatitis artefacta should always be considered when cutaneous findings are inconsistent with known dermatoses and accompanied by psychological or neurological disturbances.

## Author Contributions

**Munazza Iqbal:** writing – original draft. **Muhammad Hassaan Javaid:** data curation, writing – original draft. **Maaz ullah:** conceptualization, methodology. **Okasha Tahir:** writing – original draft. **Sadia Afridi:** resources, validation. **Tayyeb Ali:** data curation. **Ejaz Ali:** writing – review and editing. **Naveed Ahmad:** investigation, writing – original draft. **Muddassir Khalid:** writing – review and editing, supervision, project administration. **Farooq Haider:** visualization. **Fred Segawa:** resources.

## Funding

The authors have nothing to report.

## Disclosure

Patient Perspective: The patient reported that understanding the psychological basis of her skin disease helped reduce feelings of guilt and frustration. She described cognitive‐behavioral therapy as beneficial in improving emotional control and sleep quality. She expressed satisfaction with the multidisciplinary approach and reported increased confidence in preventing further self‐inflicted skin injury.

## Ethics Statement

The authors have nothing to report.

## Consent

The authors obtained written consent from patients for their photographs and medical information to be published in print and online and with the understanding that this information may be publicly available. Patient consent forms were not provided to the journal but are retained by the authors.

## Conflicts of Interest

The authors declare no conflicts of interest.

## Data Availability

Data available on request from the authors.
